# A multimodal data-based diagnostic model for predicting vestibular migraine: a retrospective study

**DOI:** 10.3389/fneur.2025.1723008

**Published:** 2025-12-03

**Authors:** Sai Zhang, ShuaiJie Yin, Shuo Qin, Yilin Lang, Wenting Wang, Shaona Liu, Ting Zhang, Shuangmei Yan, Dong Li, Yongci Hao, Ping Gu

**Affiliations:** 1 Hebei Medical University, Shijiazhuang, China; 2 Department of Neurology, The first Hospital of Hebei Medical University, Shijiazhuang, China; 3 Vertigo Center, First Hospital of Hebei Medical University, Shijiazhuang, China

**Keywords:** vestibular migraine, multimodal data, diagnostic prediction model, logistic regression, ROC curve

## Abstract

**Objective:**

Vestibular migraine (VM) is a common neurological disorder characterized by recurrent vertigo and migraine symptoms. Due to its heterogeneous clinical presentation and lack of objective biomarkers, VM is often misdiagnosed. This study aimed to develop a diagnostic prediction model for VM based on multimodal data to improve diagnostic accuracy.

**Methods:**

A total of 288 patients who visited the Vertigo Clinic of our Hospital between January 2023 and December 2024 were enrolled, including 141 VM patients and 147 non-VM controls. Multimodal data were collected, including clinical features, vestibular function tests, hematological indicators, contrast transthoracic echocardiography, and psychological assessments. Logistic regression was used to construct the prediction model, and its performance was evaluated using receiver operating characteristic (ROC) curve analysis.

**Results:**

VM patients were more likely to be female, younger, and had lower body mass index (BMI) compared to controls. They also exhibited higher rates of photophobia, phonophobia, tinnitus, emotional triggers, insomnia, and family history of migraine or vertigo. Vestibular function tests showed fewer peripheral abnormalities and more central pathway dysfunction in VM patients. Hematological analysis revealed lower levels of vitamin D and D-dimer, and higher platelet counts and calcium levels in VM patients. Right-to-left shunt (RLS) was more prevalent in VM patients. The final model included six variables: BMI, emotional triggers, insomnia triggers, history of motion sickness, and abnormal otoacoustic emissions at 8000 Hz (left ear) and 6,000 Hz (right ear). The model achieved an Area under the ROC curve of 0.8788 (95% CI: 0.8374–0.9202), indicating strong diagnostic performance.

**Conclusion:**

The multimodal diagnostic prediction model developed in this study demonstrates high preliminary accuracy. It shows potential as a clinical tool for improving the diagnosis of VM, but its generalizability requires validation in larger, prospective cohorts.

## Introduction

Vestibular migraine (VM) is a common neurological disorder characterized by recurrent episodes of dizziness or vertigo, often accompanied by headache, visual aura, nausea, and vomiting (1–3). Epidemiological studies indicate that the prevalence of VM ranges from 1 to 3%, with a significantly higher incidence in women than in men, particularly among young and middle-aged adults (4, 5). The condition substantially impairs patients’ quality of life and social functioning. Although diagnostic criteria for VM have been established jointly by the International Headache Society (IHS) and the Bárány Society (2, 6), the clinical presentation of VM is highly heterogeneous and lacks specific biomarkers.

In recent years, the rapid development of artificial intelligence and multimodal data fusion technologies has opened new avenues for medical diagnostics (7). Multimodal data integrates clinical features, imaging, laboratory tests, and functional assessments, offering a more comprehensive understanding of disease pathophysiology (8). However, existing studies on VM have mostly focused on unimodal data analysis or comparisons between VM and a single vestibular disorder, limiting the generalizability and clinical applicability of the proposed models (9, 10). The pathogenesis of VM remains incompletely understood. Current hypotheses include abnormalities in the trigeminal-vestibular pathway, cortical spreading depression, and neuroinflammatory responses (11, 12). These mechanisms suggest that multimodal data—such as vestibular function tests, hematological indicators, and contrast echocardiography—may reflect different aspects of VM pathophysiology. Therefore, developing a diagnostic prediction model based on multimodal data is of great significance for improving the accuracy and efficiency of VM diagnosis.

Although diagnostic criteria for VM have been established, its clinical diagnosis remains challenging, particularly in distinguishing it from other vestibular disorders that lack definitive biomarkers and present with overlapping symptomatology. While disorders such as benign paroxysmal positional vertigo (BPPV) and Ménière’s disease (MD) possess relatively characteristic clinical features or confirmatory tests (e.g., positional maneuvers for BPPV, audiometric findings for MD), the differential diagnosis between VM and conditions such as persistent postural-perceptual dizziness (PPPD), visual vertigo, and central vestibular disorders is often more nuanced and relies heavily on clinical interpretation. This diagnostic uncertainty underscores the need for objective tools. Therefore, this study aims to construct a diagnostic prediction model by integrating multimodal data to improve accuracy in this complex differential diagnostic landscape.

This study aims to construct an effective and practical diagnostic prediction model for VM by integrating multimodal data, including clinical characteristics, vestibular function tests, blood parameters, and right-to-left shunt (RLS) assessments. The proposed model is expected to serve as an objective and reliable tool to support clinical decision-making in the diagnosis of VM.

## Methods

### Study population

This retrospective cohort study identified 288 patients who met the inclusion criteria between January 2023 and December 2024. Data were systematically extracted from the patients’ electronic medical records, including demographic information, clinical history, diagnostic test results, and treatment details. All extracted data were de-identified prior to analysis. This study has been reviewed and approved by the Medical Ethics Committee (Approval No. [2023]00086), which granted a waiver for informed consent. Among them, 141 patients were diagnosed with VM, and 147 were classified as non-VM controls (including 90 with benign paroxysmal positional vertigo, 41 with Ménière’s disease, 13 with acute unilateral vestibulopathy, and 3 with SHL accompanied by vertigo).

### Inclusion and exclusion criteria

VM patients were diagnosed according to the criteria (International Classification of Vestibular Disorders, ICVD) jointly established by the Bárány Society and the International Classification of Headache Disorders (ICHD-3), which included: (a) at least five episodes of moderate or severe vestibular symptoms lasting 5 min to 72 h; (b) Current or past history of migraine with or without aura as defined by ICHD-3; (c) At least 50% of vestibular episodes accompanied by migrainous features (e.g., unilateral pulsating headache, photophobia, phonophobia, or visual aura); (d) Symptoms not better explained by another vestibular or ICHD disorder. The criteria for probable VM included: (a) at least five episodes of moderate or severe vestibular symptoms, each lasting from 5 min to 72 h; (b) having a history of migraine or experiencing migraine-like symptoms during the episodes; (c) these symptoms cannot be better explained by another vestibular disorder or an International Classification of Headache Disorders (ICHD) disease. Non-VM patients were diagnosed based on ICVD criteria for BPPV, MD, and AUVP (3, 13, 14), or according to the 2019 U. S. clinical practice guideline for SHL with vertigo (1). To minimize the risk of misclassification, all patient charts, particularly those with atypical features or diagnostic uncertainty, were reviewed by at least two senior neurologists/otologists. Cases where a consensus on the diagnosis could not be reached were excluded from the study.

Exclusion criteria included: comorbid vestibular disorders, severe systemic diseases (cardiac, pulmonary, hepatic, or renal), and major psychiatric or cognitive impairments.

### Data collection

#### Demographic data and clinical features

Demographic data (age, sex, BMI), clinical features (duration and frequency of attacks, accompanying symptoms), triggering factors (emotional stress, insomnia, fatigue), and medical history (motion sickness, hypertension, and diabetes) were collected. Vestibular function tests included bithermal caloric testing, video head impulse test (vHIT), cervical and ocular vestibular evoked myogenic potentials (cVEMP/oVEMP), videonystagmography (VNG), and otoacoustic emissions (OAEs). OAEs were employed to objectively assess the functional integrity of cochlear outer hair cells. This approach was chosen over pure-tone audiometry based on literature suggesting that VM may involve subclinical cochlear dysfunction detectable by OAEs prior to any shift in hearing thresholds (1, 2). While pure-tone audiometry remains the gold standard for diagnosing hearing loss, our aim was to explore finer, objective functional aspects of the auditory pathway in VM.

#### Laboratory assessments

The selection of hematological and biochemical parameters was guided by existing literature on vestibular and migraine disorders. Vitamin D was included due to its established association with vestibular pathologies such as BPPV (15, 16). At present, there is a lack of comparable evidence supporting the routine investigation of other vitamins (A, B, C, E, K) in the context of VM. Coagulation profiles and platelet indices were assessed based on emerging evidence of microthrombotic and platelet activation mechanisms in migraine and VM (17, 18). Inflammatory markers were included to explore neuroinflammatory pathways (11), while electrolytes were evaluated given the potential role of ion channel dysfunction in VM (11). All tests were part of the routine diagnostic evaluation for vertigo patients at our center and were not performed solely for research purposes.

#### Rationale for contrast transthoracic echocardiography (c-TTE)

Right-to-left shunt (RLS), often due to a patent foramen ovale (PFO), has been implicated in the pathophysiology of migraine, potentially via paradoxical embolism or bypass of pulmonary filtration of vasoactive substances (19, 20). Given the shared pathophysiological pathways between migraine and vestibular migraine (VM), and emerging evidence of a higher prevalence of RLS in patients with VM (21), we included c-TTE as an exploratory modality within our multimodal data approach. This allowed us to objectively assess the presence and magnitude of RLS and investigate its potential role as a discriminative feature in VM diagnosis.

#### Psychological assessments

Psychological assessments were conducted using the Hamilton Anxiety Scale (HAMA), Hamilton Depression Scale (HAMD), Dizziness Handicap Inventory (DHI), Pittsburgh Sleep Quality Index (PSQI), and Symptom Severity Scale (SSS).

### Variable selection for predictive model

The selection of variables for the multivariable logistic regression model was a multi-step process. First, an initial pool of candidate variables was identified based on their established clinical relevance and documented association with vestibular migraine or its differential diagnoses in the prior literature. Second, univariate analyses were performed to compare these variables between the Vestibular Migraine and control groups; variables with a *p*-value < 0.05 were retained for further consideration. Finally, these candidate variables were entered into a multivariable logistic regression model. The final, most parsimonious model was selected using a backward stepwise elimination algorithm based on the Akaike Information Criterion (AIC). This approach ensured that the final model included a robust set of predictors that are clinically plausible, statistically informative, and non-redundant.

Prior to model construction, we assessed the pattern and proportion of missing data for all candidate predictor variables identified through univariate analysis. The six variables ultimately included in the final logistic regression model (BMI, emotional triggers, insomnia triggers, history of motion sickness, abnormal left ear OAE at 8,000 Hz, and abnormal right ear OAE at 6,000 Hz) exhibited a very low rate of missingness (each <1%). Given the minimal and likely random nature of the missing data, we employed a complete-case analysis approach for the multivariable logistic regression. Consequently, any case with a missing value in one or more of these six variables was excluded from the final model fitting process. This approach ensured data integrity and simplicity without introducing significant bias due to the negligible amount of missing information.

### Statistical analysis

All analyses were performed using SPSS 26.0. Normally distributed continuous variables were expressed as mean ± standard deviation and compared using independent-sample *t*-tests. Non-normally distributed data were analyzed using the Wilcoxon rank-sum test. Categorical variables were compared using the chi-square test or Fisher’s exact test. A two-tailed *p*-value < 0.05 was considered statistically significant.

Variables with *p* < 0.01 and no missing data in univariate analysis were included as candidate predictors. Logistic regression modeling was performed using the backward elimination method, with entry and removal criteria set at *p* < 0.05. Model performance was evaluated using receiver operating characteristic (ROC) curve analysis to calculate the area under the curve (AUC). The Hosmer-Lemeshow test was used to assess model goodness-of-fit.

## Results

### Clinical characteristics

Significant differences were observed between the VM and non-VM groups in several clinical features. The VM group had a higher proportion of females, younger age, and lower BMI (*vs*. Non-VM group, *p* < 0.05). Patients with VM exhibited higher incidences of photophobia, phonophobia, tinnitus, and aural fullness, while hearing loss was less common (*vs*. Non-VM group, *p* < 0.05). The co-occurrence of dizziness and headache was more frequent in VM patients, who were also more likely to report emotional stress, insomnia, and fatigue as triggers (vs. Non-VM group, *p* < 0.05). Additionally, VM patients had higher rates of motion sickness, emotional disorders, and family history of dizziness or headache, but lower rates of hypertension, diabetes, and hyperlipidemia (vs. Non-VM group, *p* < 0.05) (Table 1).

**Table 1:** Comparison of clinical characteristics between VM and non-VM groups.

Variable	VM (*n* = 141)	Non-VM (*n* = 147)	Statistic (*Z*/*χ*^2^)	*P*-value
Gender (female), *n* (%)	117 (82.98)	98 (66.67)	10.120	0.001
Age (years), mean ± SD	45.16 ± 16.71	62.42 ± 11.72	−8.604	0.000
BMI (kg/m^2^), mean ± SD	23.89 ± 3.71	24.91 ± 3.34	−2.472	0.013
Photophobia, *n* (%)	62 (43.97)	2 (1.36)	75.604	0.000
Phonophobia, *n* (%)	64 (45.39)	3 (2.04)	75.757	0.000
Tinnitus, *n* (%)	108 (76.60)	91 (61.90)	7.274	0.007
Aural fullness, *n* (%)	115 (81.56)	104 (70.75)	4.618	0.032
Hearing loss, *n* (%)	12 (8.51)	40 (27.21)	17.010	0.000
Dizziness with headache, *n* (%)	95 (67.38)	9 (6.25)	114.859	0.000
Triggered by fatigue, *n* (%)	41 (29.08)	12 (8.16)	20.965	0.000
Triggered by emotion, *n* (%)	26 (18.44)	2 (1.36)	23.918	0.000
Triggered by insomnia, *n* (%)	19 (13.48)	2 (1.36)	15.625	0.000
Triggered by other factors, *n* (%)	37 (26.24)	17 (11.56)	10.176	0.001
Blurred vision, *n* (%)	19 (13.48)	5 (3.40)	9.561	0.002
Other visual symptoms, *n* (%)	20 (14.18)	3 (2.04)	14.443	0.000
Motion intolerance, *n* (%)	89 (63.57)	92 (63.01)	0.010	0.922
Nausea, *n* (%)	36 (25.53)	36 (24.49)	0.042	0.838
Vomiting, *n* (%)	74 (52.48)	69 (46.94)	0.885	0.347
History of motion sickness, *n* (%)	63 (44.68)	4 (2.72)	70.979	0.000
History of emotional disorders, *n* (%)	64 (45.39)	36 (24.49)	13.870	0.000
History of hypertension, *n* (%)	31 (21.99)	67 (45.58)	17.844	0.000
History of diabetes, *n* (%)	8 (5.67)	31 (21.09)	14.606	0.000
History of hyperlipidemia, *n* (%)	12 (8.51)	13 (8.84)	0.010	0.920
History of coronary heart disease, *n* (%)	9 (6.38)	9 (6.12)	0.008	0.927
History of cerebral infarction, *n* (%)	3 (2.13)	10 (6.80)	3.649	0.056
Family history of dizziness, *n* (%)	10 (7.09)	1 (0.68)	7.989	0.005
Family history of headache, *n* (%)	23 (16.31)	0 (0.00)	26.060	0.000

BMI, Body Mass Index; n, number of cases; SD, standard deviation.

### Vestibular function test results

Comprehensive vestibular function testing revealed distinct profiles between the VM and non-VM groups. VM patients demonstrated a significantly lower prevalence of abnormalities across all tested frequencies of otoacoustic emissions (OAEs) in both ears (all *p* < 0.001), as detailed in Table 2.

**Table 2:** Comparison of vestibular function between VM and non-VM groups.

Variable	VM (*n* = 141)	Non-VM (*n* = 147)	Statistic (*Z*/*χ*^2^)	*P*-value
Left ear OAE 750 Hz abnormal, *n* (%)	60 (42.55)	95 (64.63)	14.108	0.000
Left ear OAE 1 kHz abnormal, *n* (%)	46 (32.62)	91 (61.90)	24.740	0.000
Left ear OAE 1.5 kHz abnormal, *n* (%)	39 (27.66)	71 (48.30)	12.987	0.000
Left ear OAE 2 kHz abnormal, *n* (%)	29 (20.57)	72 (48.98)	25.514	0.000
Left ear OAE 3 kHz abnormal, *n* (%)	29 (20.57)	76 (51.70)	30.112	0.000
Left ear OAE 4 kHz abnormal, *n* (%)	36 (25.53)	83 (56.46)	28.397	0.000
Left ear OAE 6 kHz abnormal, *n* (%)	51 (36.17)	106 (72.11)	37.487	0.000
Left ear OAE 8 kHz abnormal, *n* (%)	69 (48.94)	118 (80.27)	31.035	0.000
Right ear OAE 750 Hz abnormal, *n* (%)	62 (43.97)	98 (66.67)	15.013	0.000
Right ear OAE 1 kHz abnormal, *n* (%)	47 (33.33)	88 (59.86)	20.342	0.000
Right ear OAE 1.5 kHz abnormal, *n* (%)	35 (24.82)	75 (51.02)	20.924	0.000
Right ear OAE 2 kHz abnormal, *n* (%)	31 (21.99)	70 (47.62)	20.767	0.000
Right ear OAE 3 kHz abnormal, *n* (%)	25 (17.73)	66 (44.90)	24.576	0.000
Right ear OAE 4 kHz abnormal, *n* (%)	32 (22.70)	82 (55.78)	32.946	0.000
Right ear OAE 6 kHz abnormal, *n* (%)	46 (32.62)	100 (68.03)	36.089	0.000
Right ear OAE 8 kHz abnormal, *n* (%)	74 (52.48)	112 (76.19)	17.685	0.000
Spontaneous nystagmus positive, *n* (%)	29 (20.57)	47 (32.41)	5.142	0.023
Bithermal caloric intolerance, *n* (%)	32 (22.70)	15 (11.11)	6.550	0.010
Post-head-shaking nystagmus positive, *n* (%)	14 (29.79)	13 (92.86)	17.392	0.000
Supine position nystagmus positive, *n* (%)	5 (10.87)	19 (13.01)	0.147	0.701
Left lateral position nystagmus positive, *n* (%)	7 (15.91)	41 (27.89)	2.584	0.108
Right lateral position nystagmus positive, *n* (%)	7 (15.91)	40 (27.21)	2.332	0.127
Left head-hanging nystagmus positive, *n* (%)	4 (9.09)	40 (27.21)	6.271	0.012
Right head-hanging nystagmus positive, *n* (%)	8 (18.18)	42 (28.57)	1.892	0.169
Spontaneous nystagmus intensity (°/s), mean ± SD	0.83 ± 2.30	1.30 ± 2.66	−2.221	0.026
Caloric nystagmus intensity (°/s), mean ± SD	0.93 ± 3.29	1.52 ± 3.52	−3.175	0.001
CP (%), mean ± SD	19.61 ± 18.36	25.56 ± 21.57	−2.188	0.029
DP (%), mean ± SD	15.85 ± 14.93	22.29 ± 21.08	−2.417	0.016
Saccade abnormality, *n* (%)	10 (7.09)	4 (2.72)	2.973	0.085
Pursuit abnormality, *n* (%)	8 (5.67)	12 (8.16)	0.690	0.406
Optokinetic nystagmus abnormality, *n* (%)	2 (1.42)	6 (4.08)	—	0.283
cVEMP asymmetry ratio, mean ± SD	0.14 ± 0.13	0.18 ± 0.12	2.427	0.015
oVEMP asymmetry ratio, mean ± SD	0.15 ± 0.11	0.17 ± 0.14	0.997	0.319
vHIT gain reduction, *n* (%)	7 (5.22)	19 (14.84)	6.777	0.009
vHIT saccade positivity, *n* (%)	73 (54.48)	84 (66.14)	3.701	0.054
LHC gain value, mean ± SD	1.05 ± 0.14	1.02 ± 0.18	−1.83	0.067
RHC gain value, mean ± SD	1.13 ± 0.75	1.06 ± 0.19	−0.709	0.478
LAC gain value, mean ± SD	1.09 ± 0.17	1.12 ± 0.19	−1.239	0.217
RAC gain value, mean ± SD	1.28 ± 0.20	1.27 ± 0.23	−0.675	0.499
LPC gain value, mean ± SD	1.24 ± 0.21	1.18 ± 0.23	2.337	0.020
RPC gain value, mean ± SD	1.02 ± 0.16	0.96 ± 0.17	−2.357	0.018

OAE, otoacoustic emission; cVEMP, cervical vestibular evoked myogenic potential; oVEMP, ocular vestibular evoked myogenic potential; vHIT, video head impulse test; LHC, left horizontal canal; RHC, right horizontal canal; LAC, left anterior canal; RAC, right anterior canal; LPC, left posterior canal; RPC, right posterior canal; CP, canal paresis; DP, directional preponderance.

Regarding oculomotor and vestibular reflex function, the VM group had a lower incidence of spontaneous nystagmus (20.57% vs. 32.41%, *p* = 0.023) but a higher incidence of bithermal caloric intolerance (22.70% vs. 11.11%, *p* = 0.010). Quantitative analyses showed that the VM group exhibited lower intensities of both spontaneous nystagmus (0.83 ± 2.30 °/s vs. 1.30 ± 2.66 °/s, *p* = 0.026) and caloric-induced nystagmus (0.93 ± 3.29 °/s vs. 1.52 ± 3.52 °/s, *p* = 0.001). Furthermore, the VM group showed significantly lower rates of post-head-shaking nystagmus (29.79% vs. 92.86%, *p* < 0.001) and nystagmus in the left Dix-Hallpike position (9.09% vs. 27.21%, *p* = 0.012). The mean canal paresis (CP) value (19.61% ± 18.36% vs. 25.56% ± 21.57%, *p* = 0.029) and directional preponderance (DP) value (15.85% ± 14.93% vs. 22.29% ± 21.08%, *p* = 0.016) were also significantly lower in the VM group.

Assessment of vestibular evoked myogenic potentials indicated a lower cVEMP asymmetry ratio in the VM group (0.14 ± 0.13 vs. 0.18 ± 0.12, *p* = 0.015). The video head impulse test (vHIT) revealed a lower prevalence of gain reduction in the VM group (5.22% vs. 14.84%, *p* = 0.009). Interestingly, the VM group presented with higher vHIT gain values for the left posterior canal (LPC, 1.24 ± 0.21 vs. 1.18 ± 0.23, *p* = 0.020) and the right posterior canal (RPC, 1.02 ± 0.16 vs. 0.96 ± 0.17, *p* = 0.018). No significant inter-group differences were observed for other vHIT canals, oVEMP asymmetry ratio, or the prevalence of saccadic intrusions.

### Scale assessment results

VM patients scored significantly higher on the HAMA (anxiety) and SSS (somatization) scales compared to the non-VM group (*p* < 0.05), suggesting a higher prevalence of anxiety and somatic symptoms. No significant differences were found in HAMD (depression), PSQI (sleep quality), or DHI (dizziness handicap) scores between the two groups (*p* > 0.05) (Table 3).

**Table 3:** Comparison of scale scores between VM and non-VM patient groups.

Variable	VM (*n* = 141)	Non-VM (*n* = 147)	Statistic (*Z*)	*P*-value
HAMA, mean ± SD	11.96 ± 5.79	9.14 ± 4.00	−2.37	0.018
HAMD, mean ± SD	14.21 ± 6.87	11.56 ± 4.90	−1.805	0.071
SCL-90, mean ± SD	162.02 ± 55.11	144.81 ± 42.24	−1.132	0.257
SSS, mean ± SD	42.40 ± 12.08	36.77 ± 9.04	−2.191	0.028
PSQI, mean ± SD	9.43 ± 4.96	9.87 ± 4.06	0.441	0.659
DHI-total, mean ± SD	30.08 ± 12.67	31.19 ± 11.59	0.4	0.689
DHI-functional, mean ± SD	13.62 ± 5.15	13.78 ± 5.24	0.211	0.833
DHI-emotional, mean ± SD	7.01 ± 4.54	7.78 ± 5.75	0.361	0.718
DHI-physical, mean ± SD	9.53 ± 5.08	10.00 ± 4.80	0.505	0.613

HAMA, Hamilton Anxiety Scale; HAMD, Hamilton Depression Scale; SCL-90, Symptom Checklist-90; SSS, Symptom Severity Scale; PSQI, Pittsburgh Sleep Quality Index; DHI = Dizziness Handicap Inventory.

### Hematological and biochemical indicators

The VM patients had significantly lower levels of vitamin D, D-dimer, APTT ratio, PT activity, white blood cell count, neutrophil percentage, platelet distribution width, magnesium, and potassium, while showing higher levels of APTT, PT, international normalized ratio (INR), lymphocyte percentage, eosinophil percentage, platelet count, plateletcrit, and calcium (*p* < 0.05). No significant differences were observed in cytokine levels (e.g., IL-6, TNF-*α*) between the two groups (*p* > 0.05) (Table 4).

**Table 4:** Comparison of hematological indicators between VM and non-VM patient groups.

Variable	VM (*n* = 141)	Non-VM (*n* = 147)	Statistic (T/Z)	*P*-value
IL-1β (pg/mL), mean ± SD	3.08 ± 4.39	2.18 ± 2.26	−0.841	0.400
IL-2 (pg/mL), mean ± SD	1.92 ± 1.42	1.65 ± 1.41	−1.432	0.152
IL-4 (pg/mL), mean ± SD	1.52 ± 1.46	1.40 ± 1.58	−0.742	0.458
IL-5 (pg/mL), mean ± SD	1.38 ± 1.03	2.07 ± 3.67	0.244	0.807
IL-6 (pg/mL), mean ± SD	7.65 ± 12.25	7.62 ± 9.27	1.049	0.294
IL-8 (pg/mL), mean ± SD	39.55 ± 92.05	69.09 ± 122.81	1.607	0.108
IL-10 (pg/mL), mean ± SD	3.48 ± 5.95	2.87 ± 2.01	−0.090	0.928
TNF-α (pg/mL), mean ± SD	5.84 ± 6.77	10.08 ± 26.24	−0.504	0.614
IFN-γ (pg/mL), mean ± SD	2.14 ± 2.09	1.64 ± 1.24	−0.906	0.365
IL-17A (pg/mL), mean ± SD	3.91 ± 4.90	2.83 ± 3.47	−1.563	0.118
IL-12P70 (pg/mL), mean ± SD	1.74 ± 1.55	1.36 ± 0.97	−1.057	0.291
IFN-α (pg/mL), mean ± SD	2.21 ± 2.25	1.81 ± 2.60	−0.923	0.356
Vitamin D (ng/mL), mean ± SD	17.12 ± 7.93	19.53 ± 6.70	−3.13	0.002
D-dimer (mg/L), mean ± SD	0.33 ± 0.48	0.49 ± 0.99	−3.10	0.002
Thrombin time (s), mean ± SD	14.76 ± 0.94	14.87 ± 1.40	−0.091	0.927
Fibrinogen (g/L), mean ± SD	2.88 ± 0.55	2.97 ± 0.56	−0.989	0.323
APTT (s), mean ± SD	30.35 ± 3.02	28.91 ± 2.82	4.168	0.000
APTT ratio, mean ± SD	1.06 ± 0.11	1.02 ± 0.13	3.782	0.000
PT (s), mean ± SD	11.18 ± 0.68	11.07 ± 1.19	−2.460	0.014
PT activity (%), mean ± SD	99.68 ± 9.39	102.33 ± 11.86	2.551	0.011
INR, mean ± SD	1.01 ± 0.06	1.00 ± 0.11	−2.586	0.010
WBC count (×10^9^/L), mean ± SD	6.00 ± 2.03	6.39 ± 1.69	−2.648	0.008
Neutrophil percentage (%), mean ± SD	56.48 ± 13.09	62.64 ± 11.91	−4.398	0.000
Monocyte percentage (%), mean ± SD	7.12 ± 2.27	6.85 ± 2.82	1.326	0.185
Lymphocyte percentage (%), mean ± SD	33.17 ± 10.94	28.30 ± 10.23	4.317	0.000
Eosinophil percentage (%), mean ± SD	1.96 ± 1.83	1.59 ± 1.51	1.993	0.046
Basophil percentage (%), mean ± SD	0.53 ± 0.37	0.48 ± 0.39	1.381	0.167
Hemoglobin (g/L), mean ± SD	129.45 ± 18.34	133.48 ± 14.29	−1.856	0.064
Platelet count (×10^9^/L), mean ± SD	247.83 ± 62.69	225.92 ± 65.66	3.085	0.002
Mean platelet volume (fL), mean ± SD	8.78 ± 1.36	8.90 ± 1.14	−1.453	0.146
Plateletcrit (%), mean ± SD	0.21 ± 0.05	0.20 ± 0.05	3.056	0.002
Platelet distribution width (%), mean ± SD	16.51 ± 0.48	16.79 ± 1.28	−2.241	0.025
Calcium (mmol/L), mean ± SD	2.29 ± 0.12	2.27 ± 0.10	2.008	0.046
Magnesium (mmol/L), mean ± SD	0.87 ± 0.07	0.89 ± 0.07	−2.371	0.018
Potassium (mmol/L), mean ± SD	3.86 ± 0.34	3.87 ± 0.31	−0.192	0.848
Phosphorus (mmol/L), mean ± SD	1.22 ± 0.25	1.14 ± 0.21	2.903	0.004

IL, interleukin; TNF-α, tumor necrosis factor-alpha; IFN, interferon; APTT, activated partial thromboplastin time; PT, prothrombin time; INR, international normalized ratio; WBC, white blood cell.

### Contrast echocardiography findings

The VM group had a significantly higher prevalence and grade of RLS detected by c-TTE compared to the non-VM group (*p* < 0.05), suggesting a potential association between RLS and VM pathogenesis (Table 5).

**Table 5:** Differences in RLS shunt volume between VM and non-VM patient groups.

Variable	VM (*n* = 137)	Non-VM (*n* = 38)	Statistic (*χ*^2^)	*P*-value
RLS Grade			8.275	0.041
Grade 0	45 (32.85)	22 (57.89)		
Grade I	21 (15.33)	4 (10.53)		
Grade II	25 (18.25)	3 (7.89)		
Grade III	46 (33.58)	9 (23.68)		

RLS, right-to-left shunt; Grade 0 = no microbubbles; Grade I = <10 microbubbles/frame; Grade II = 10–30 microbubbles/frame; Grade III = > 30 microbubbles/frame.

### Model construction and evaluation

**Figure 1:**
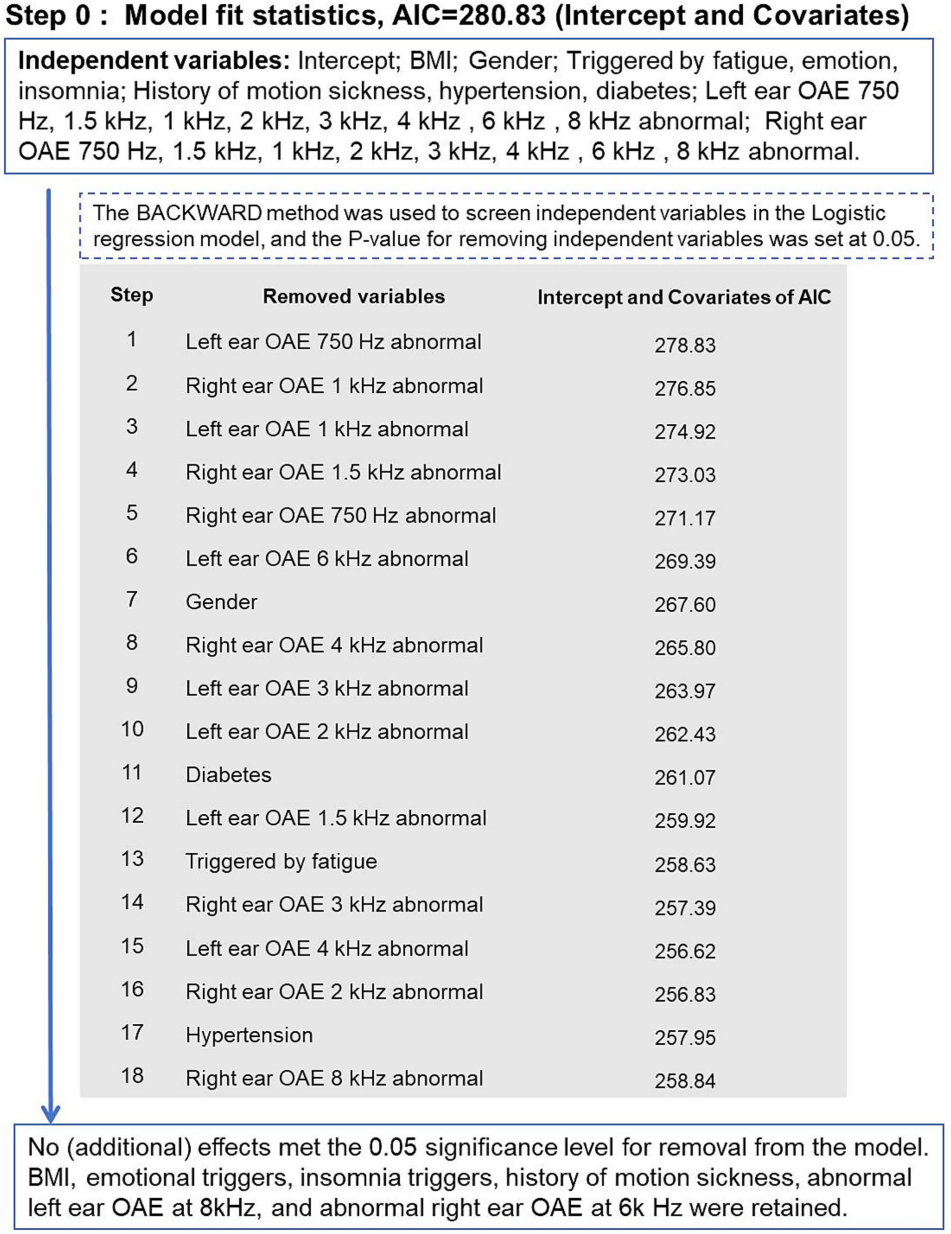
The process of building the predictive model.

After considering clinical relevance, data completeness, and accessibility, six variables were included in the final logistic regression model: BMI, emotional triggers, insomnia triggers, history of motion sickness, abnormal left ear OAE at 8000 Hz, and abnormal right ear OAE at 6000 Hz. The Hosmer-Lemeshow goodness-of-fit test indicated that the model was well calibrated (χ^2^ = 11.8106, *p* = 0.1599) (Figure 1).

**Figure 2:**
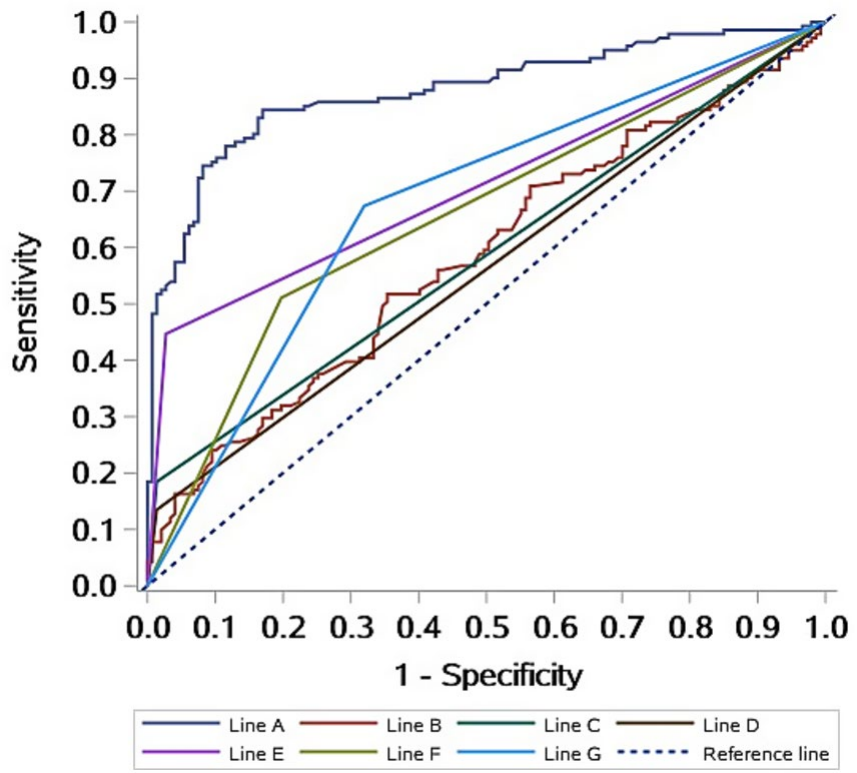
ROC curves of the diagnostic prediction model for VM patients. Line A: Logistic model; Line B: BMI; Line C: Emotional triggers; Line D: insomnia triggers; Line E: History of motion sickness; Line F: Abnormal left ear OAE at 8,000 Hz; Line G: Abnormal right ear OAE at 6,000 Hz.

The ROC curve analysis demonstrated that the model had strong discriminative ability for diagnosing VM, with an AUC of 0.8788 (95% CI: 0.8374–0.9202). This suggests that the model has high accuracy in distinguishing VM patients from non-VM individuals (Figure 2). These results indicate that the multimodal prediction model holds strong potential for clinical application.

## Discussion

The VM is a major cause of recurrent spontaneous vertigo, yet its recognition remains poor. In this study we integrated multimodal data—demographic variables, triggers, comorbidities, vestibular test results, hematological indices and RLS status—to develop a parsimonious diagnostic prediction model. After univariate logistic regression and backward elimination, six routinely collected variables—BMI, mood trigger, insomnia trigger, history of motion sickness, abnormal left 8 kHz OAE and abnormal right 6 kHz OAE—were retained. The AUC was 0.8788, indicating excellent discriminative performance.

The lifetime prevalence of VM is 0.98% and the 1-year prevalence 0.89% (22), yet misdiagnosis rates of 30–70% have been reported (23, 24). The clinical phenotype is heterogeneous and overlaps with benign paroxysmal positional vertigo, Menière’s disease, persistent postural-perceptual dizziness and central causes of vertigo. Current IHS diagnostic criteria rely on expert interpretation of historical features, and no laboratory or imaging biomarker is available. Consequently, diagnosis remains clinician-dependent and error-prone. Multimodal data fusion has proven valuable in other neurological disorders and is now gaining traction in neuro-otology (25, 26). We combined phenotypic, vestibular, inflammatory, hematological and cardiac shunt variables to capture the multidimensional nature of VM pathophysiology. BMI entered the final model with an inverse association: each 1 kg/m^2^ increment reduced the odds of VM by 10.3%. This is counter-intuitive given previous reports linking obesity to migraine, but may reflect selection bias in our modest sample or true biological divergence between migraine with and without vestibular aura. Adiposity modulates neuro-inflammation, insulin resistance and trigeminovascular activation, yet VM may preferentially affect lean individuals with heightened central vestibular excitability (27). The initially observed lower prevalence of metabolic comorbidities such as hypertension, diabetes, and hyperlipidemia in VM patients appears to be a consequence of these demographic differences. Therefore, these factors should not be considered protective elements specific to VM, but rather reflective of the typical demographic profile of the migraine population from which VM patients are drawn. This underscores the importance of controlling for fundamental demographic variables when comparing disease cohorts.

Mood and sleep disturbances were strong predictors. VM patients report a 2–3-fold higher prevalence of anxiety and insomnia than those with cerebellar pathology (28). Functional imaging studies implicate dysregulation of serotonergic thalamocortical circuits, heightened insular and limbic reactivity, and altered sleep micro-architecture (29, 30). These data support a bidirectional relationship in which affective and sleep disorders both trigger and perpetuate VM attacks. Motion-sickness susceptibility, present in half of VM patients (31–33), reflects impaired multisensory integration within temporo-parieto-insular vestibular networks (34). Central, rather than peripheral, vestibular dysfunction predominates in VM, consistent with the lower frequency of peripheral OAE failure observed in this cohort.

The prevalence and intensity of spontaneous nystagmus, the intensity of positional nystagmus in the supine position, the prevalence of post-head-shaking nystagmus, the prevalence of nystagmus in the left head-hanging position, the CP value, the DP value, the cVEMP asymmetry ratio, and the prevalence of left cVEMP abnormality, as well as the gain reduction rate in vHIT, were all lower in VM patients than in non-VM patients. This suggests that the proportion of VM patients with peripheral vestibular organ damage is relatively low, which is related to the types of diseases in the non-VM group. In this study, the prevalence of bithermal caloric intolerance was significantly higher in the VM group than in the non-VM group, which is considered to be related to the hyperactivation of brain regions such as the insular cortex, thalamus, frontal lobe, and limbic lobe in VM patients (35, 36).

The HAMA and SSS scores of VM patients were significantly higher than those of non-VM patients, which is consistent with previous study that found a high comorbidity of emotional disorders (37). However, no significant differences were found between the two groups in HAMD, PSQI, and DHI scores. These results suggest that the emotional disorders in VM patients are mainly characterized by anxiety, which is consistent with the findings of Beh et al. (31). This may be related to the frequent attacks of the disease and the disease burden caused by the associated autonomic nervous system symptoms.

Inflammatory markers, D-dimer and platelet indices differed between groups but were excluded from the final model because of missing data. Pro-inflammatory cytokines such as TNF-α and IFN-γ may activate trigeminovascular pathways, yet their elevation is non-specific, occurring also in Menière’s disease and affective disorders (38, 39). Larger prospective studies with standardized sampling and multiplex assays are required to clarify the role of immunity in VM. Some studies suggest that the pathogenesis of VM is associated with mechanisms such as microthrombi, platelet activation, and ion channel dysfunction (17, 18, 40). In this study, analysis of the data from the enrolled patients also revealed differences between VM and non-VM patients in hematological indicators such as platelet count and D-dimer levels. While several hematological indices differed between groups in univariate analysis, their exploratory nature and exclusion from the final model indicate that these associations are preliminary. They generate hypotheses for future research into inflammatory and coagulatory pathways in VM but should not be considered diagnostic.

The RLS has previously been of interest in migraine patients due to paradoxical embolism. Since many of the pathophysiological mechanisms of VM are derived from migraine, an increasing number of scholars are now paying attention to the impact of RLS on VM or the therapeutic effects of RLS intervention on VM (41). In this study, the prevalence and volume of RLS were significantly higher in the VM group than in the control group, which is consistent with previous research findings (41, 42). Expanding the sample size of the study and refining the classification and grading of RLS will help to clarify the specific relationship between RLS and the occurrence of VM and identify VM patients who may benefit from surgical intervention.

By integrating multimodal data, we are able to more comprehensively capture the pathological features of VM, thereby enhancing diagnostic accuracy. In this study, we constructed a diagnostic prediction model for VM using logistic regression and assessed the model’s predictive performance via the ROC. The results indicated that the prediction model fits well and has high predictive efficacy, effectively distinguishing between VM and non-VM patients. Moreover, the six indicators that ultimately entered the model, including BMI, are all easily obtainable, which also increases the applicability and generalizability of the model in clinical practice.

In this initial model-development study, we selected a control group comprising the most prevalent diagnoses in our vertigo clinic (BPPV, MD, AUVP, etc.) to establish a foundational model capable of distinguishing VM from a broad range of common peripheral vestibular disorders. Given the number of candidate variables relative to the sample size, the findings of this study, including the identified predictors and the model itself, are primarily hypothesis-generating and necessitate confirmation in larger, independent datasets.

Despite the achievements of this study, there are still some limitations. First, the relatively small sample size may affect the universality of the results. Future studies can increase the sample size to further improve the accuracy and reliability of the model. Adding validation sets to extrapolate the model’s applicability is also recommended. In addition, this study only used logistic regression models. In the future, other more advanced machine learning algorithms, such as deep learning models, can be attempted to further enhance the model’s performance. Furthermore, our control group consisted of patients with other vestibular disorders, which, while reflecting the real-world diagnostic challenge, carries a potential risk of diagnostic contamination given the known phenotypic overlap between VM and conditions such as Ménière’s disease. Although we employed stringent diagnostic criteria to mitigate this, the inclusion of a healthy control cohort in future prospective studies would be essential to further validate the specificity of the identified predictive features and to distinguish them from general vestibulopathy.

In conclusion, we have developed a pragmatic, multimodal diagnostic model that distinguishes VM from other causes of vertigo with high accuracy. Integration of BMI, affective triggers, sleep disturbance, motion-sickness history and high-frequency OAE abnormalities offers clinicians an evidence-based tool to reduce misdiagnosis. Prospective multicenter validation and refinement using deep learning are warranted to enhance applicability across global health-care settings.

## Data Availability

The raw data supporting the conclusions of this article will be made available by the authors, without undue reservation.
